# Prevalence and antimicrobial resistance patterns of *Clostridium perfringens* from healthy broiler chickens: A potential public health threat

**DOI:** 10.1016/j.onehlt.2025.101156

**Published:** 2025-07-30

**Authors:** Tsepo Ramatla, Silence Ncube, Prudent Mokgokong, Jane Nkhebenyane, Lesego Molale-Tom, Rendani Ndou, Ntelekwane Khasapane, Carlos Bezuidenhout, Oriel Thekisoe, Kgaugelo Lekota

**Affiliations:** a Centre for Applied Food Safety and Biotechnology, Department of Life Sciences, Central University of Technology, 1 Park Road, Bloemfontein 9300, South Africa; b Unit for Environmental Sciences and Management, North-West University, Potchefstroom 2531, South Africa; c Department of Animal Health, School of Agriculture, North-West University, Mmabatho 2735, South Africa

**Keywords:** *C. perfringens*, Prevalence, Toxins, Broiler chicken, Antibiotic resistance

## Abstract

The aim of this study was to investigate the prevalence and toxin type of *Clostridium perfringens* isolated from broiler chicken faeces and determine its antibiotic resistance (AR) profile. A total of 480 broiler chicken faeces were collected from four different chicken abattoirs in North West Province, South Africa. Faecal samples were pooled (5 per pool from the same farm), resulting in 96 pooled samples. The disc diffusion method was used for documenting phenotypic AR, whilst PCR was used for the identification of *C. perfringens* and detection of toxin and antibiotic resistance genes. All 52 isolates identified as *Clostridium* spp. using the *tpi* gene PCR assay were also positive for the *16S rRNA* gene which is specific for *C. perfringens*. All 52*C. perfringens* isolates harboured the *cpa* gene responsible for encoding alpha toxin. Additionally, 7 (13.5 %) of these isolates were found to carry the *netB* gene. None of the isolates harboured *cpe*, *cpb2*, and *cpb* genes. All isolates in this study exhibited AR to ampicillin, followed by tetracycline, clindamycin, and chloramphenicol with resistance rates of 100 %, 71.15 %, 46.15 %, and 34.62 %, respectively. *C. perfringens* isolates contained tetracycline encoding genes, namely *tet*(A) and *tet*(W), chloramphenicol encoding genes which are: *flo*R, *cat*I, and *cat*II and beta-lactamase encoding genes including *bla*_TEM_, *bla*_SHV_, *bla*_CTX-M_ and *bla*_OXA_. None of the isolates carried *bla*_CARB_. This is the first study to characterize *C. perfringens* and determine its antimicrobial susceptibility phenotypically and genetically in food-producing chicken in South Africa, proving that animals may be sources of resistant strains of *C. perfringens*.

## 1 Background

*Clostridium* spp. are anaerobic, Gram-positive, spore-forming rod-shaped bacterium that can produce endospores commonly found in soil, sewage, soil, food, faeces and water [[1], [2], [3], [4]]. The genus *Clostridium* comprises 181 identified species, divided into 16 clusters based on the *16S rRNA* gene phylogeny, highlighting their considerable genetic diversity [5,6]. *Clostridium* species are found in various human and animal sites, including the intestinal tract, female genital tract, and oral mucosa [[7], [8], [9]]. However, among the clostridial species, *Clostridium perfringens* is known to cause pleuropulmonary infections [7]. Furthermore, *C. perfringens* is a pathogen of considerable clinical concern, given its role in causing serious human diseases [6,10,11].

*C. perfringens* naturally inhabits the gastrointestinal tracts of poultry, cattle, sheep, and goats [12,13]. Due to concerns about food-borne intoxication, *C. perfringens* is an organism of high zoonotic potential and of serious public health concern [14]. *C. perfringens* is a prolific toxin-producer, harboring toxin genes on its chromosome and plasmids [5]. It produces at least 20 toxic enzymes and spore-forming anaerobic bacteria [15]. Based on the production of four primary toxins, namely alpha (*cpa*), beta (*cpb*), epsilon (*etx*), and iota (*iap*), *C. perfringens* strains are divided into five toxin categories which are designated type A to E [2,3,13]. Epsilon toxin, a Category B Select Agent, is produced by *C. perfringens* types B and D. Although there is a potential threat, there is currently no evidence of human infection or epsilon-intoxication [11]. Human food poisoning strains carrying enterotoxigenic *C. perfringens* type A carry *cpe* on the chromosome, which is clustered into the transposon-like structure Tn1565 flanked by insertion sequences IS1470 and IS1469 [13]. Alpha toxin significantly contributes to the virulence of *C. perfringens*, causing gas gangrene, haemolysis, and septicaemia in humans, as demonstrated by in vitro and in vivo animal studies [16].

Antibiotic resistance is a rising public health crisis and one of the greatest health problems of the twenty-first century [17,18]. Various antimicrobial agents have been used to treat infections caused by *C. perfringens*, including tetracycline, chloramphenicol, lincomycin, bacitracin, ampicillin, metronidazole, and even imipenem [19]. Several resistance genes have been discovered in *C. perfringens* isolates thus far [17,19]. Although these antibiotics boost growth and feed efficiency, they alter gut flora and put pressure on the development of antibiotic resistance [17,20].

Despite the simplicity of preventing *C. perfringens* foodborne illnesses through proper washing and disinfection, significant outbreaks with occasional fatal consequences continue to occur [21]. Chicken remains the most affordable meat alternative, playing a vital role in ensuring household food and nutrition security, as well as the stability of the South African food system [22]. *C. perfringens* is one of leading cause of foodborne illness outbreaks in South Africa [22]. Therefore, this study aimed to investigate the prevalence, toxin type and antibiotic resistance profiles of *C. perfringens* from faecal samples of broiler chickens.

## 2 Materials and methods

### 2.1 Sampling

A total of 480 faecal samples were aseptically collected from caeca/rectum of healthy broiler chicken's post-evisceration. They then transferred to sterile containers and stored them in cooler boxes for transportation to the laboratory. Pooled samples were created from 480 faecal samples, resulting in a total of 96 samples with five samples per pool [23,24].

### 2.2 Isolation and identification of *C. perfringens*

A modified version of the Fung double tube method was used to analyze each sample. A capped test Pyrex tube was filled with 7 mL of double-strength *C. perfringens* agar base (Oxoid, UK) and autoclaved. Liquefied media was cooled to approximately 50 °C, 1 mL of sample and 32 μL of TSC supplement (Oxoid, UK) were mixed with the agar in serial dilutions. The mixture was then transferred to a Pyrex test tube containing an autoclaved inserter test tube, sealed to create anaerobic conditions.

The test tube was incubated at 37 °C for 3–7 h to inhibit excessive bacterial growth. Black colonies formed in the center of the media. Isolation of *Clostridium* colonies was done by pouring the contents of the tube into a sterile petri dish, and the colonies were collected with a sterile wooden toothpick. These colonies were then sub-cultured in Luria-Bertani (LB) plates in an AnaeroJar (AG0025; Oxoid), with an AnaeroGen sachet (AN0025; Thermo Scientific) and an anaerobic indicator (BR0055B; Oxoid).

### 2.3 DNA extraction and identification of Clostridium species by PCR

Genomic DNA was extracted from pure bacterial cultures using PureLink® DNA Mini Kit (Invitrogen, USA) following the manufacturer's instructions. A NanoDrop spectrophotometer (ThermoFischer Scientific, USA) was used to measure DNA concentrations. To detect the *tpi* gene, a PCR assay was used to amplify an 800 bp gene fragment with tpi-F and tpi-R primers (Table 1) with conditions described by Montso and Ateba [6]. The positive control was *C. perfringens* ATCC 13124 (ThermoFischer Scientific™), while the negative control was nuclease-free water.

**Table 1:** Antibiotic resistance gene primers and PCR conditions used in this study.

Name of bacteria	Target gene	Primer	Primer sequence (5′ → 3′)	Amplicon size (bp)	Annealing temp (°C)	References
	***Clostridium* species identification**					
*Clostridium* species	*tpi*	tpi-F tpi-R	GCWGGWAAYTGGAARATGMAYAA TTWCCWGTWCCDATWGCCCADAT	800	60	[6]
*C. perfringens*	*16S RNA*	ClPer-1 ClPer-2	TAACCTGCCTCATAGAGT TTTCACATCCCACTTAATC	481	53	[14]
	**Toxins**					
Alpha (*α*)	*cpa*	CPAlpha-F CPAlpha-R	GCTAATGTTACTGCCGTTGA CCTCTGATACATCGTGTAAG	195	53	[14]
Beta (*β*)	*cpb*	CPBeta-F3 CPBeta-R3	GCGAATATGCTGAATCATCTAG GCAGGAACATTAGTATATCTTC	548	53	[14]
Beta-2 (*β*2)	*cpb2*	CPBeta2-F2 CPBeta2-R2	AAATATGATCCTAACCAACAA CCAAATACTCTAATYGATGC	548	53	[25]
Enterotoxin	*cpe*	CPEntero-F CPEntero-R	TTCAGTTGGATTTACTTCTG TGTCCAGTAGCTGTAATTT	485	53	[14]
netB toxin	*netB*	NETB-F NETB-R	TGATACCGCTTCACATAAAGGTTGG ATAAGTTTCAGGCCATTTCATTTTTCCG	196	61	[26]

### 2.4 Identification of *C. perfringens*

*C. perfringens* was identified using the ClPer-1 and ClPer-2 oligonucleotide primers (Table 1) to detect *C. perfringens* with slight modification from previously published protocol by Rana et al. [14]. The *16S rRNA* gene sequence was used to confirm the identity of the *C. perfringens* isolates which were positive by the above CIPer primers PCR assay. To amplify the *16S rRNA* gene segment, bacterial universal primers (27F: AGA GTT TGA TCM TGG CTC AG and 1492R: GGT TAC CTT GTT ACG ACT T) were used the following methods described by Mlangeni et al. [27]. PCR conditions were as follows: 96 °C initial denaturation for 4 min, followed by 30 cycles of denaturation at 94 °C for 30 s, annealing at 57 °C for 30 s and extension at 72 °C for 1 min, and an extension step of 10 min at 72 °C. The amplified *16S rRNA* gene fragments were sequenced with the BigDye Terminator cycle sequencing kit (v 3.1) on the SeqStudio genetic analyzer at North-West University, UESM Sequencing facility in Potchefstroom. The representative sequences were analyzed using BLASTn (https://blast.ncbi.nlm.nih.gov/Blast.cgi) to verify the isolate's identity.

### 2.5 Detection of *C. perfringens* toxin genes

All positive isolates were chosen for the screening of four toxin genes [*netB* (necrotic enteritis-β-like toxin), *cpe* (Enterotoxin), *cpb2* (*β*2), *cpa* (α), and *cpb* (*β*)] upon identification of the *C. perfringens* species (Table 1). Multiplex PCR was used to amplify the toxinotyping gene specifically, as described in a prior study. For the PCR assays detecting virulence genes, a total of 25 μL reaction mixture consisting of 12.5 μL of the PCR Master Mix [AmpliTaq Gold® DNA Polymerase, 0.05 units/L, Gold buffer, 930 mM Tris/HCl pH 8.05, 100 mM KCl, 0.4 mM of each dNTP, and 5 mM MgCl_2_].

(AmpliTaq Gold® DNA Polymerase), 10 μM of each primer, 2 μL of template DNA, and 8.5 μL nuclease-free water. Amplification was performed in the thermal cycler, the ProFlex PCR System (Applied Biosystems, USA), with the PCR program consisting of 15 min 95 °C followed by 40 cycles of 30 s denaturation at 94 °C, 90 s annealing [53 °C - 61 °C] and 90 s extension at 72 °C and a final extension step of 10 min at 72 °C (Table 1).

### 2.6 Agarose gel electrophoresis

PCR products were analyzed on 1.5 % (*w*/*v*) ethidium bromide-stained agarose gels and visualized under UV light using the ENDURO GDS Gel Documentation System (Labnet International Inc., US). Product sizes were determined using 100 bp and 1 kb DNA ladders (PROMEGA, Wisconsin, USA).

### 2.7 Phenotypic antibiotic resistance screening

All 52*C. perfringens* were screened for AMR. Five antibiotics, namely, Ampicillin (AMP), Clindamycin (CLI), Chloramphenicol (CHL), Metronidazole (MTZ) and Tetracycline (TET) obtained from ThermoFischer Scientific™ were tested on anaerobic bacteria using minimum inhibitory concentration (MIC) gradient diffusion (M.I.C.E. strips) [28] and agar dilution [29], which is considered the gold standard method for testing anaerobic bacteria. In the agar dilution, molten Reinforced Clostridia agar was supplemented with antibiotics of varying concentrations, ranging from 0.015 μg/mL to 256 μg/mL. Afterwards, the mixture was poured into sterile petri dishes and allowed to set. A different concentration of antibiotics was spot inoculated onto each plate and incubated anaerobically at 37 °C for 24 to 48 h. The MIC breakpoints were interpreted according to the CLSI Interpretive Standards for Anaerobes [30,31]. As previously reported, multidrug resistance (MDR) was determined as a phenomenon that occurs with resistance to three or more classes of antimicrobial agents [32]. *C. perfringens* ATCC 13124 (ThermoFischer Scientific™) served as the quality control strain.

### 2.8 Detection of antibiotic resistance genes by PCR

The genomic DNA extracted from *C. perfringens* isolates was screened for the presence of antibiotic resistance genes encoding for chloramphenicol (*catI, catII, catIII, catIV*, and *floR*), tetracycline [*tet(A), tet(O), tet(X), tet(P), tet(W)* and *tet(K*)], *β*-lactamase (*bla*_SHV_*, bla*_OXA_*, bla*_CARB_*, bla*_TEM_ and *bla*_CTX-M_) (Table 2). To set up PCR assays for the AMR genes, a total of 25 μL reaction mixture consisting of 12.5 μL of the PCR Master Mix (AmpliTaq Gold® DNA Polymerase, 0.05 units/L, Gold buffer, 930 mM Tris/HCl pH 8.05, 100 mM KCl, 0.4 mM of each dNTP, and 5 mM MgCl2), 10 μM of each primer, 2 μL of template DNA, and 8.5 μL nuclease-free water. The amplification program consisted of the following steps: denaturation at 94 °C for 6 min, followed by 30 cycles of 94 °C for 30 s, annealing temperature listed in Table 2 for 30 s, and 72 °C for 60 s, with a final extension at 72 °C for 6 min [33].

**Table 2:** Antibiotic resistance gene primers used in this study.

Target gene	Primers	Primer sequence (5′ → 3′)	Amplicon size (bp)	Annealing temp (°C)
**Tetracycline**				
*tet*(A)	TETA-F TETA-R	GCGCTNTATGCGTTGATGCA ACAGCCCGTCAGGAAATT	387	62
*tet*(O)	TETO-F TETO-R	ACGGARAGTTTATTGTATACC TGGCGTATCTATAATGTTGAC	171	60
*tet*(W)	TETW-F TETW-R	GAGAGCCTGCTATATGCCAGC GGGCGTATCCACAATGTTAAC	168	50
*tet*(K)	*tet*(X)-F *tet*(X)-R	TCGATAGGAACAGCAGTA CAGCAGATCCTACTCCTT	169	61
**Chloramphenicol**				
*cat*I	catI-F catI-R	GGTGATATGGGATAGTGTT CCATCACATACTGCATGATG	349	60
*cat*II	catII-F catII-R	GATTGACCTGAATACCTGGAA CCATCACATACTGCATGATG	567	60
*cat*III	catIII-F CatIII-R	CCATACTCATCCGATATTGA CCATCACATACTGCATGATG	275	60
*cat*IV	CatIV-F catIV R	CCGGTAAAGCGAAATTGTAT CCATCACATACTGCATGATG	451	60
*flo*R	FloR-F FloR-R	CGCCGTCATTCCTCACCTTC GATCACGGGCCACGCTGTGTC	215	50
***β*-lactamase**				
*bla*_SHV_	SHV-F SHV-R	CACTCAAGGATGTATTGT G TTAGCGTTGCCAGTGCTCG	885	55
*bla*_OXA_	OXA-F OXA -R	ACACAATACATATCAACTTCGC AGTGTGTTTAGAATGGTGATC	813	55
*bla*_CARB_	CARB-F CARB-R	CAAGTACTTTYAAAACAATAGC GCTGTAATACTCCKAGCAC	534	46
*bla*_TEM_	TEM-F TEM-R	TTCTTGAAGACGAAAGGGC ACGCTCAGTGGAACGAAAAC	1150	55
*bla*_CTX-M_	CTX-M-F CTX-M-R	GTTACAATGTGTGAGAAGCAG CCGTTTCCGCTATTACAAAC	550	55

### 2.9 Data analysis

The *16S rRNA* sequences from four representative *C. perfringens* isolates were aligned with nucleotide sequences available in the National Centre for Biotechnology Information database (NCBI) GenBank using BLASTn (http://www.ncbi.nlm.nih.gov/BLAST/). Heatmap plots illustrating the virulence and antibiotic resistance profiles were generated using ChipPlot, an online tool accessible at https://www.chiplot.online/. Data management and all statistical analyses were done using Microsoft Excel version 2506 (One Microsoft Way, USA). Statistical analysis of normally distributed data was performed using one-way analysis of variance (ANOVA), followed by Tukey's post hoc test to determine significant differences.

## 3 Results

### 3.1 Prevalence and toxin-encoding genes of *C. perfringens* from broiler chickens

**Fig. 1:**
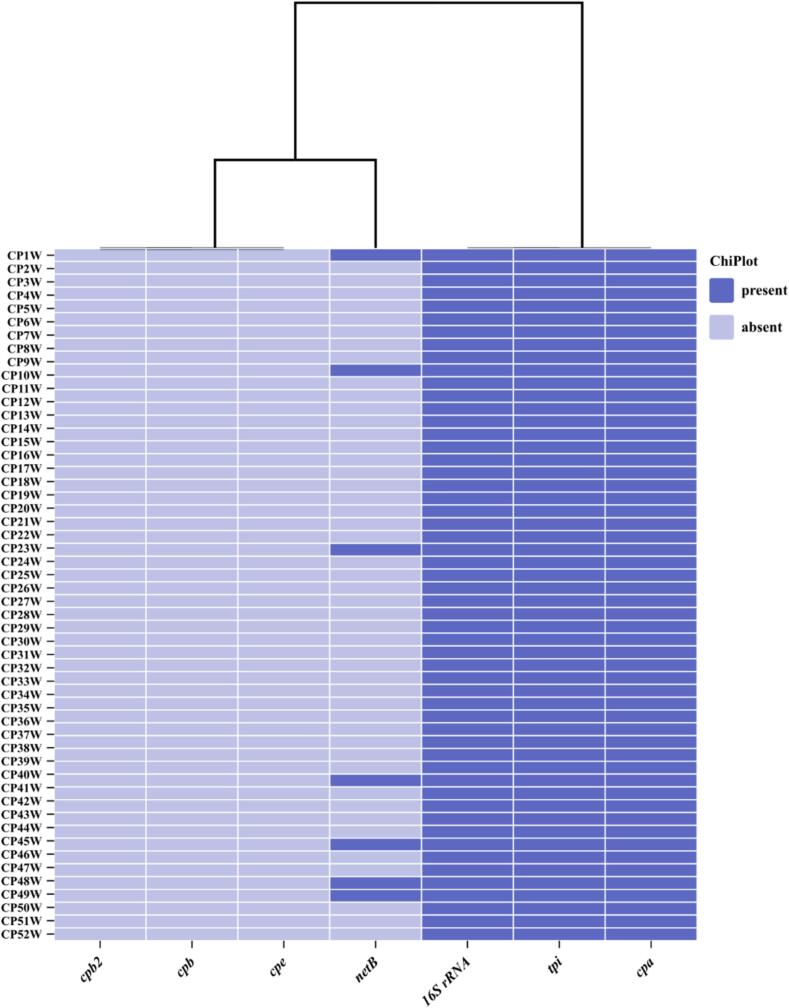
Heatmap showing confirmatory and virulent genes detected in 52*C. perfringens* isolates from broiler chickens. The black colour indicates the genes that were detected in each isolate. https://www.chiplot.online/#.

A total of 96 pooled broiler samples were analyzed for the presence of *C. perfringens* by the conventional cultural characteristic's method. From each positive plate, two colonies were picked and sub-cultured. A total of 52 isolates were confirmed to be *Clostridium* spp. by *tpi* gene PCR assay (Fig. 1). The *16S rRNA* gene sequence analysis of the *C. perfringens* revealed a high percentage of nucleotide similarity (99.9–100 %) to the reference GenBank sequences of the *C. perfringens* isolates. The representative isolates were deposited in GenBank with the following accession numbers: OR494052, OR494053, OR494054 and OR494055. Furthermore, all 52*C. perfringens* encode the species-specific (*cpa*) gene for *C. perfringens* (Fig. 1). The *netB* gene was detected in 7 (13.5 %) of the 52*C. perfringens* isolates examined. None of the *cpb*, *cpe*, and *cpb2* genes were detected in all screened isolates.

Table 3 shows the number of 52*C. perfringens*-positive samples per slaughterhouse. Among all positive samples, Abattoir B has the highest number (16/52; 30.8 %) of positive isolates, followed by abattoir D with 14 (26.9 %), then Abattoir A with 13 (25 %) isolates and finally abattoir C with only 9 (17.3 %) isolates.

**Table 3:** The number of samples collected per abattoir and *C. perfringens* positive samples.

Abattoirs ID	Samples collected	Pooled samples	*C. perfringens* encoding genes			Toxin encoding genes			
			*tpi* (%)	16S RNA (%)	Alpha (α) [*cpa*] (%)	Beta (β) [*cpb*] (%)	Beta-2 (*β*2) [*cpb2*] (%)	Enterotoxin [*cpe*] (%)	netB toxin [*netB*] (%)
**A**	120	24	25	25	13.5	0	0	0	3.8
**B**	120	24	30.7	30.7	21.1	0	0	0	5.7
**C**	120	24	17.3	17.3	5.7	0	0	0	0
**D**	120	24	26.9	26.9	9.6	0	0	0	23.8
***p*-value**	–	–	0.74	0.74	0.01	–	–	–	–

### 3.2 Antimicrobial resistance of *C. perfringens* strains

**Fig. 2:**
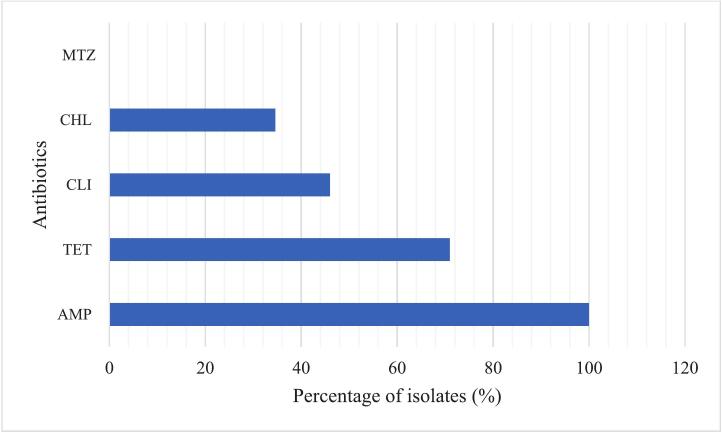
Distribution of antibiotic resistance among *C. perfringens*. The abbreviation refers to: AMP = Ampicillin, CLI=Clindamycin, CHL = Chloramphenicol, MTZ = Metronidazole, and TET = Tetracycline.

All *C. perfringens* isolates (*n* = 52) were susceptible to metronidazole, as shown in Fig. 2. All the isolates obtained in this study 100 % (*n* = 52) were resistant to ampicillin, followed by tetracycline, clindamycin, and chloramphenicol, with 71.15 % (*n* = 37), 46.15 % (*n* = 24), and 34.62 % (*n* = 18), respectively. Of the 52 isolates, 24 (44.4 %) exhibited multidrug resistance, showing resistance to ≥3 antibiotic classes.

### 3.3 Identification of tetracycline, chloramphenicol and β-lactamase encoded genes

**Fig. 3:**
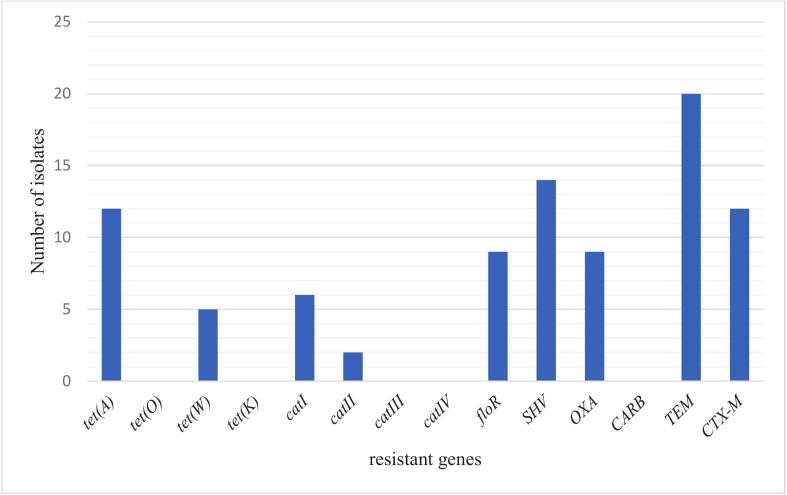
Distribution of antibiotic-resistant genes among *C. perfringens* isolated from healthy broiler chickens.

The tetracycline encoding genes were identified in 13 (25 %) isolates, which harboured *tet*(A) and *tet*(W) 5 (9.6 %) genes. None of the isolates carried *tet*(K) and *tet*(O) genes. Whereas chloramphenicol encoding genes such as for *flo*R, *cat*I and *cat*II from 9 (17.3 %), 6 (11.5 %) and 2 (3.8 %) were detected from screened *C. perfringens* in this study, respectively. Whilst *cat*III and *cat*IV were not detected from all the isolates. Out of 52 ampicillin phenotypic resistant isolates, only 21 (40.4 %), 15 (28.8 %), 12 (23.1 %) and 9 (17.3 %) harboured *bla*_TEM_, *bla*_CTX-M_, *bla*_SHV_*,*and *bla*_OXA_ respectively, which are genes encoding beta-lactamase. Five (CP1W, CP12W, CP20W, CP29W and CP38W) isolates possess up to three classes of antibiotics (tetracycline, chloramphenicol and *β*-lactamase). No *tet*(O), *tet*(K), *cat*III, *cat*IV, *bla*_CARB_ and *cat*IV genes detected in all the tested isolates (Fig. 3).

## 4 Discussion

Livestock and humans are susceptible to *C. perfringens* infections, which cause intestinal infections and histotoxic diseases [34]. *C. perfringens* is a ubiquitous commensal bacterium that resides in the gastrointestinal tracts of humans and animals [35]. Faecal flora contamination of carcasses is unavoidable during slaughter. Therefore, food of animal origin may serve as a medium for transporting resistant bacteria, including *C. perfringens,* between animals and man [36]. Globally, *C. perfringens* is one of the most common bacteria that cause foodborne illness [37]. There is a paucity of studies in South Africa on the isolation of *C. perfringens* in water sources [28], captive animals [38], human [39] and beef samples [6]. However, there is a scarcity of investigations regarding the prevalence of *C. perfringens* in chickens in South Africa.

This study revealed that 27 % (26/96) of the pooled chicken faecal samples harboured *C. perfringens*, which is higher than the 23.4 % of chicken faecal samples reported in Beijing and Shanxi, China [40], and in central China (23.1 %) [41], in Korea, 19 % from chicken, beef and pork samples [42], 16 % isolates from chicken meat in Vietnam [1] and 17.4 % isolates from wild bird stool samples in Beijing, China [4]. However, the *C. perfringens* prevalence in this study was lower (38.42 %) than that reported in a study conducted by Xu et al. [43], in China and 56.7 % of the isolates from aquatic sources in China [44]. This indicates regional variations in prevalence and potential differences in sampling methods, biosecurity measures, or environmental factors that influence *C. perfringens* contamination. Further investigation into these contributing factors could aid in controlling and mitigating its spread in poultry and related environments.

*C. perfringens* toxinotypes correlate with distinct disease syndromes, with alpha-toxin (encoded by *cpa*) found in nearly all toxinotypes [45]. In 2018, the introduction of *cpe* and *netB* genes as novel typing markers led to the identification of two new toxinotypes (F and G) distinct from toxinotype A, increasing the total number of recognized toxinotypes in *C. perfringens* to seven (A-G) [46]. *C. perfringens* type A, which produces the single major toxin CPA, serves as the foundational toxinotype for this species. Acquisition of plasmids encoding specific toxins, including *C. perfringens* enterotoxin, *C. perfringens* epsilon toxin, *C. perfringens* iota-toxin, and necrotic enteritis toxin B-like toxin, confers distinct toxinotypes on *C. perfringens* strains [47]. The *cpa* breaks down phosphatidylcholine and sphingomyelin on the cell membrane, inhibits neutrophil migration and maturation, and activates arachidonic acid metabolism, causing vasoconstriction and platelet aggregation [48]. Therefore, this toxin impairs innate immunity and creates an undesirable micro-environment [49]. In the present study, all *C. perfringens* harboured *cpa* of healthy broiler chicken’ samples. The production of NetB toxin has recently been recognized as a key virulence factor in *C. perfringens* strains that induce necrotic enteritis (NE). The prevalence of NetB is greater in diseased birds, yet it is also found in healthy broilers [50]. In this study, the *netB* gene was detected in 7 (13.5 %) of the 52*C. perfringens* isolates examined. Zhou et al. [51] determined that although *netB* is essential for NE virulence, it cannot independently induce NE and requires additional genes for complete virulence. However, none of the isolates in this study possessed Enterotoxin (*cpe*), and Beta-toxin (*cpb2* and *cpb*) genes.

In the present study, all *C. perfringens* isolates (100 %) were resistant to ampicillin, followed by tetracycline, clindamycin, and chloramphenicol, with 71.15 %, 46.15 %, and 34.62 %, respectively. In an Iranian study, *C. perfringens* isolates from raw beef meats showed high antibiotic resistance to ampicillin, tetracycline, amoxicillin, ciprofloxacin and chloramphenicol antibiotics with 72.2 %, 66.6 %, 61.1 %, 37.8 % and 33.3 %, respectively [47]. A study in India on *C. perfringens* isolates from livestock and poultry found resistance rates of 44 % to gentamicin, 40 % to both erythromycin and bacitracin, and 26.6 % to tetracycline [52]. A Romanian study by Beres et al. [19] found high antibiotic resistance rates among *C. perfringens* isolates from food-producing animals, with 71.4 % resistant to tetracycline, 64.2 % to penicillin, 42.8 % to erythromycin, and 35.7 % to enrofloxacin. A consensus among numerous studies indicates that *C. perfringens* isolates frequently exhibit resistance to tetracycline [1]. Due to excessive usage and incorrect veterinary guidance, *C. perfringens* isolates have developed resistance to tetracycline [19,47]. In Vietnam, 91.30 % of pork and chicken meat isolates were resistant to tetracycline [1]. Among antibiotics, tetracyclines are the second most used group after *β*-Lactam [53]. Tetracycline resistance is achieved through the presence of one or more of the 36 known *tet* genes [28]. This study revealed that 44.38 % of the *C. perfringens* isolates exhibited multidrug resistance, showing resistance to ≥3 antibiotic classes. In contrast, a study recently conducted in Iran by Hassani et al. [47] showed lower multidrug resistance (38 %) prevalence of *C. perfringens* isolates compared to our results.

Globally, antimicrobial resistance is a significant concern [17]. Even though these antibiotics boost growth and feed efficiency, they alter gut flora and pressure antibiotic resistance development [17,20]. Through conjugative plasmids, ARG may spread between different bacterial infections [47]. In this study, we found the presence of tetracycline-encoding genes like *tet*(A) (25 %) and *tet*(W) (9.6 %) genes. At least two [*tet*(W) and *tet*(A)] tetracycline resistance genes were present in each of the 13 tetracycline-resistant isolates. Remarkably, the combinations of tetracycline(disk) and tetracycline encoding genes *tet*(A) and *tet* (W) were found in one sample.

Out of 18*C. perfringens* isolates resistant to chloramphenicol, seven had one chloramphenicol encoding genes *catI*, *catII* and *floR* in this study. One *C. perfringens* isolate had phenotypic resistance to chloramphenicol and harboured three chloramphenicol encoding genes (*catI*, *catII* and *floR*). All CAT enzymes can acetylate chloramphenicol to form 3-acetoxy-chloramphenicol [29]. The *β*-Lactam antibiotics are medicine and agriculture's most commonly used antibiotics [28]. This study identified the presence of genes encoding beta-lactamases, including *bla*_OXA_, *bla*_CTX-M_, *bla*_SHV_*,* and *bla*_TEM_. The detection of these genes in *C. perfringens* poses a significant public health concern, as they can be released into the environment, disseminated among other microorganisms, and potentially contribute to the emergence of antibiotic-resistant superbugs. Due to the quantity and variety of human antibiotics used in veterinary clinics, the findings raised serious concerns for public health and veterinary medicine.

## 5 Conclusion

This study provides insights into the prevalence, toxinotypes, and antibiotic resistance patterns of *C. perfringens* isolates obtained from broiler chickens in South Africa. The study confirms that all isolates were identified as *C. perfringens*, harboring the *cpa* and *netB* genes which has previously been reported to play a crucial role in necrotic enteritis in poultry. Therefore, ongoing year-round monitoring of *Clostridium* disease in broiler chickens is essential. The isolates showed high resistance to tetracycline, clindamycin, and chloramphenicol. Different classes of ARGs were detected in this study. In broiler farming, the occurrence of multiple ARGs could contribute to development of pathogenic multidrug-resistant *C. perfringens* strains. Given the role of foodborne transmission in spreading *C. perfringens*, stricter biosecurity measures, surveillance programs, and antimicrobial stewardship are urgently needed to mitigate its impact.

## CRediT authorship contribution statement

**Tsepo Ramatla:** Writing – original draft, Methodology, Formal analysis, Data curation, Conceptualization. **Silence Ncube:** Investigation, Formal analysis. **Prudent Mokgokong:** Writing – review & editing, Methodology. **Jane Nkhebenyane:** Writing – review & editing. **Lesego Molale-Tom:** Writing – review & editing. **Rendani Ndou:** Writing – review & editing. **Ntelekwane Khasapane:** Writing – review & editing. **Carlos Bezuidenhout:** Writing – review & editing, Supervision. **Oriel Thekisoe:** Writing – review & editing, Supervision, Funding acquisition, Conceptualization. **Kgaugelo Lekota:** Writing – review & editing, Supervision, Conceptualization.

## Ethics approval and consent to participate

The ethical approval was received from the Ethical Committee of the Animal Production Committee of the North-West University, South Africa (NWU-00511-18-A5).

## Funding

This research was funded by NRF Incentive grant for rated researchers (GUN: 118949) made available to Oriel Thekisoe.

## Declaration of competing interest

The authors declare that they have no known competing financial interests or personal relationships that could have appeared to influence the work reported in this paper.

## Data Availability

All the data supporting our findings were incorporated within the manuscript.
